# Distal Renal Tubular Acidosis in Sjögren's Syndrome: A Case Report

**DOI:** 10.7759/cureus.10962

**Published:** 2020-10-15

**Authors:** Scarlet Louis-Jean, Patrick R Ching, Allison Wallingford

**Affiliations:** 1 Department of Medicine, American University of Antigua, Saint John's, ATG; 2 Department of Medicine, University of Maryland Medical Center Midtown Campus, Baltimore, USA

**Keywords:** sjögren’s syndrome, renal tubular acidosis, hypokalemic paralysis

## Abstract

Sjögren’s syndrome is an autoimmune lymphocytic infiltrative disease that leads to chronic inflammatory and degradatory changes to exocrine glands and extra-glandular systemic organs. It rarely affects children and adolescents. In cases where adolescents are affected, a paucity of sicca symptoms, xerostomia, and xerophthalmia often leads to a missed diagnosis. Consequently, the first presenting sign of Sjögren’s syndrome in adolescents may be heterogeneous, with varying clinical symptoms related to parotitis or systemic organ involvement. In this case report, we discuss a 19-year-old girl with distal renal tubular acidosis (RTA), who had experienced severe hypokalemic episodes since the age of 14 years; the patient was eventually diagnosed with Sjögren’s syndrome. She was managed and maintained on potassium and alkali repletion therapy.

## Introduction

Renal tubular acidosis (RTA) is characterized by renal tubular impairment in balancing physiologic acid-base. It often results from a defect in tubular transporters, which participate in the secretion or uptake of specific ions, due to congenital causes, exposure to nephrotoxic drugs, diuretic abuse, autoimmune disease, or malignancy (e.g., multiple myeloma). There are three major types of RTA: distal or type 1, proximal or type 2, and hyperkalemic or type 4. All three types of RTA are characterized by a positive urine anion gap, hyperchloremic non-anion gap metabolic acidosis, alkalotic or acidotic urine pH, and serum potassium derangements (hypo- or hyperkalemia). 

Distal RTA (type 1 or classic RTA), which is the focus of this case report, can be further defined by an alkalotic urinary pH (>5.5) and profound hypokalemia (<3 mmol/L). It is often caused by an impairment in hydrogen ion secretion in the distal renal alpha-intercalated cells. Consequently, due to an impaired luminal gradient, ionic wasting occurs, leading to the possible development of nephrocalcinosis, nephrolithiasis, rickets/osteomalacia, muscle weakness, and respiratory failure. Distal RTA can be a rare complication of Sjögren’s syndrome in adolescents, although studies have demonstrated that approximately a greater proportion of adult patients develop defects in distal tubular acidification due to tubulointerstitial nephritis [1]. In this report, we discuss a case of distal RTA secondary to Sjögren’s syndrome in an adolescent patient and her management with potassium and alkali repletion.

## Case presentation

A 19-year-old girl with a remote history of bipolar disorder and a five-year history of multiple hospitalizations for hypokalemic paralysis was brought to the emergency room for sudden-onset bilateral thigh cramping progressing to the shoulder and biceps after awakening from a nap. The patient reported that her symptoms were similar to her previous episodes and were exacerbated by her menses, which she had recently completed four days prior to symptom onset. A review of systems did not reveal syncope, gastrointestinal disturbances, urinary symptoms, joint pain, rashes, diuretic/laxative abuse, or suicidal or homicidal ideation. There was no family history of autoimmune diseases. She endorsed smoking cannabis occasionally and denied tobacco or alcohol use. Aside from her potassium chloride and sodium bicarbonate regimen, the patient was not on any psychotropic or other medications.

Except for bradycardia (heart rate of 50 bpm), her vital signs were within normal limits and physical examination was unremarkable. Results of initial lab tests showed sodium of 143 mmol/L, potassium of 2.1 mmol/L, chloride of 110 mmol/L, and bicarbonate of 17 mmol/L. Venous blood gas pH was 7.21. Urine sodium was 74 mmol/L, urine potassium was 18.4 mmol/L, and urine chloride was 68 mmol/L. Urinalysis showed a urinary pH of 7.0 without blood or protein (Table 1). The renal sonogram did not show nephrolithiasis. Electrocardiogram showed sinus bradycardia, delayed intraventricular conduction, and U waves. With a positive urine anion gap of 24 and hyperchloremic non-anion gap metabolic acidosis, she was diagnosed with distal RTA. She was promptly hydrated with intravenous normal saline and was given potassium chloride and sodium bicarbonate, which corrected both her hypokalemia and hyperchloremic non-anion gap metabolic acidosis. She was later discharged on oral potassium chloride and sodium bicarbonate.

**Table 1:** Results of the patient's diagnostic labs ANA: antinuclear antibody; anti-dsDNA: anti-double-stranded DNA antibody; anti-SSA: anti-Sjögren’s syndrome-related antigen A; anti-SSB: anti-Sjögren’s syndrome-related antigen B; anti-U1-RNP: anti-U1 ribonucleoprotein antibody

Laboratories	Values	Reference ranges
Serum chemistry		
Sodium	143.0	135.0–145.0 mmol/L
Potassium	2.1	3.5–5.0 mmol/L
Chloride	110.0	95.0–105.0 mmol/L
Bicarbonate	17.0	12.0–22.0 mmol/L
Urine electrolytes		
pH	7.0	4.5–7.8
Sodium	74.0	40.0–220.0 mmol/L
Potassium	18.4	25.0–125.0 mmol/L
Chloride	68.0	14.0–50.0 mmol/L
Anion gap	24.0	<10 mEq/L
Venous blood gas		
pH	7.21	7.31–7.41
Autoimmune panel		
ANA	1:2560	<1:80
Anti-dsDNA	Negative	<20.0 AU/mL
Anti-Smith	Negative	0–40.0 AU/mL
Anti-Ro/SSA-52	261.0	0–40.0 AU/mL
Anti-Ro/SSA-60	130.0	0–40.0 AU/mL
Anti-La/SSB	Negative	0–40.0 AU/mL
Anti-U1-RNP	Negative	040.0 AU/mL

Over the next few months, the patient had several similar presentations at hospitals for upper and lower extremity weakness and paralysis secondary to hypokalemia. Initially, exposure to synthetic cannabinoids was believed to have induced the patient’s severe hypokalemia and acid-base disturbances in the absence of known nephrotoxic drug exposure and autoimmune history. Additionally, suspicion of disordered eating/exercise or diuretic/laxative abuse as a contributor to her metabolic derangements was low based on patient report and chart review. On further probing, she admitted to infrequent, intermittent dry mouth and dry eyes with a sand-like sensation associated with ocular pruritus for several months, but denied any history of eye inflammation, use of artificial teardrops, or increased occurrence of cavities.

Her autoimmune panel was positive for antinuclear antibodies (ANA) (1:2560) and anti-Ro/SSA antibody (SSA-52: 261 AU/mL and SSA-60: 130 AU/mL) and negative for anti-double-stranded DNA (dsDNA), anti-Smith, anti-La/SSB, and anti-U1-ribonucleoprotein (RNP) antibodies (Table 1). Diagnostic tests for SCN4A deletion/duplication, urine organic acids, plasma amino acids, carnitine (total and free), acylcarnitine, and urine porphyrins were all within normal reference values. Given the patient did not have other derangements of urine and serum electrolytes, other causes of renal tubulopathies, such as Bartter, Gitelman, and Fanconi syndromes were subsequently ruled out. Based on sicca symptoms and positive anti-Ro/SSA antibodies, a presumptive diagnosis of Sjögren’s syndrome was made. She was advised to follow up with nephrology and rheumatology in the outpatient setting; however, the patient never attended her appointments.

## Discussion

Sjögren’s syndrome is an autoimmune exocrinopathy with variable systemic involvement caused by monolymphocytic infiltration. Systemic associations can include cardiac involvement leading to prolonged QT, pulmonary disease, neurologic involvement, and tubulointerstitial disease (e.g., tubulointerstitial nephritis, RTA, Fanconi syndrome, and glomerulonephritis). However, the true mechanism behind RTA is not well known. Available data point to reduced hydrogen electrolyte secretion consequently due to immune-mediated injury of the hydrogen-ATPase pump of the alpha-intercalated cells or autoantibody directed against carbonic anhydrase II [2].

There are two classifications of the disease: primary and secondary Sjögren’s syndrome. Primary Sjögren’s syndrome is thought to be an isolated condition not often caused by another pathological process, whereas secondary Sjögren’s syndrome is a sequela of other rheumatological diseases like systemic lupus erythematosus, rheumatoid arthritis, or scleroderma. Sjögren’s syndrome often affects middle-aged women, with a sex-adjusted prevalence of 2.2-10.3 per 10,000 individuals [3]. It is often diagnosed based on the American-European Consensus Group’s (AECG) criteria for Sjögren’s syndrome, which details ocular symptom/sign(s) of three months' duration, oral symptom/sign(s), lymphocytic sialadenitis on minor salivary gland biopsy, and auto-antibodies (anti-Ro/SSA and -La/SSB) as the basis for a diagnosis [4,5]. The diagnosis of primary Sjögren’s syndrome requires patients to meet four of the six criteria, which must include histopathology or autoantibodies, or any three of four objective criteria [4]. However, only 22% of patients have been reported to meet the AECG or American College of Rheumatology's (ACR) criteria for diagnosis as the required tests are not often performed in clinical practice [3]. Moreover, the current diagnostic criteria have been shown to have limited scope in juvenile cases, thereby posing a diagnostic challenge in adolescents who present heterogeneously and do not have classical sicca symptoms [6,7]. 

Recurrent parotid swelling has been documented as the most common presenting feature in the pediatric population, occurring more frequently than sicca symptoms [8]. The most common renal manifestation of Sjögren’s syndrome is tubulointerstitial nephritis, which may present as RTA in adult populations; however, RTA is a rare occurrence in the pediatric population as it accounts for 7.1-19.2% of cases who present with renal potassium wasting or hypokalemic paralysis [9]. Our patient first presented with an episode of muscle weakness and syncope in the setting of severe hypokalemia at the age of 14 years, often exacerbated by menses. In a study by Sandhya et al., adequate levels of estrogen and androgens were associated with increased protection of glandular epithelial cells from apoptosis and lymphocytic infiltration [10,11]. Therefore, the mechanism whereby decreased levels of sex hormones lead to lymphocytic tubulopathy may explain an association with the patient’s hypokalemic exacerbation during menses especially since she endorsed increased intake of her potassium pills during her menstrual cycles.

Over a period of five years, our patient was hospitalized for several episodes of symptoms related to severe hypokalemia secondary to distal RTA. With every hospitalization, the opportunity to assess the patient for Sjögren’s syndrome was missed, likely due to a lower index of suspicion based on age and absence of classical signs and symptoms at the time. Pediatric patients with Sjögren’s syndrome have been observed to develop sicca symptoms with increasing age, which may explain why our patient endorsed infrequent intermittent pruritic dry eyes and difficulty chewing crackers during a later hospitalization [12]. Nonetheless, once obtained, autoimmune antibodies for Sjögren’s syndrome demonstrated high serum ANA and anti-Ro/SSA-52 and -60, suggesting a higher suspicion for the disease, especially as SSA-52 has a prevalence rate of 63.2% in Sjögren’s syndrome [13]. The presence of high anti-Ro/SSA titers has also been associated with a greater likelihood of earlier disease onset and extra-glandular involvement [10,14,15].

Due to a confluence of social issues that involved a contentious relationship with her parents, the patient did not follow up on her scheduled outpatient appointments. Attempts by social workers to improve the patient’s access to medical appointments were to no avail. If the patient had undergone additional diagnostic studies, glucocorticoid therapy could have been initiated. However, while glucocorticoid therapy is the universal treatment modality for patients with Sjögren’s-induced renal involvement, a review of the literature has demonstrated the recurrence of hypokalemia and acidosis with glucocorticoid tapering, especially in cases where early treatment was not started [6,1,16]. As curative treatments remain to be elucidated, monitoring for complications such as nephrocalcinosis, nephrolithiasis, and rickets/osteomalacia should also take place along with ongoing treatment of metabolic derangements and symptoms.

## Conclusions

The early identification and diagnosis of Sjögren’s syndrome can be a difficult challenge as current diagnostic guidelines contribute to a higher likelihood of missed diagnosis in pediatric cases. Juvenile patients may present with heterogeneous findings inconsistent with the classic presentation of the disease. Thus, adolescent patients presenting with evidence of renal pathologies should undergo further diagnostic investigations to rule out Sjögren’s syndrome while simultaneously receiving prompt management of metabolic derangements with potassium and alkali treatment to prevent potentially fatal sequelae.
